# Antiphospholipid antibodies and the risk of thrombosis in myeloproliferative neoplasms

**DOI:** 10.1515/biol-2022-0545

**Published:** 2023-02-07

**Authors:** Rūta Dambrauskienė, Rolandas Gerbutavičius, Milda Rudžianskienė, Renata Paukštaitienė, Astra Vitkauskienė, Erika Skrodenienė, Diana Remeikienė, Inga Zaborienė, Elona Juozaitytė

**Affiliations:** Department of Oncology and Hematology, Institute of Oncology, Lithuanian University of Health Sciences, Eivenių Str. 2, Kaunas, LT-50009, Lithuania; Department of Physics, Mathematics and Biophysics, Lithuanian University of Health Sciences, Eivenių Str. 2, Kaunas, LT-50009, Lithuania; Department of Laboratory Medicine, Lithuanian University of Health Sciences, Eivenių Str. 2, Kaunas, LT-50009, Lithuania; Department of Radiology, Lithuanian University of Health Sciences, Eivenių Str. 2, Kaunas, LT-50009, Lithuania; Department of Oncology and Hematology, Institute of Oncology, Lithuanian University of Health Sciences, Eivenių Str. 2, Kaunas, LT-50009, Lithuania

**Keywords:** myeloproliferative neoplasia, thrombosis, antiphospholipid antibodies, platelet polymorphisms

## Abstract

The morbidity and mortality of *BCR-ABL*-negative myeloproliferative neoplasia (MPN) patients is highly dependent on thrombosis that may be affected by antiphospholipid antibodies (aPLA) and *lupus* anticoagulant. Our aim was to evaluate the association of the aPLA together with platelet receptor glycoprotein (GP) Ia/IIa c.807C>T CT/TT genotypes and thrombotic complications in patients with MPNs. The study included 108 patients with *BCR-ABL*-negative MPN with data of previous thrombosis. Two different screening and one confirmatory test for the *lupus* anticoagulant were performed. Thrombotic complications were present in 59 (54.6%) subjects. aPLA were more frequently found in MPN patients with thrombosis vs no thrombosis (25.4 and 6.1%; *p* = 0.007). MPN patients with arterial thrombosis were more frequently positive for aPLA vs no arterial thrombosis (38.8 and 11.9%; *p* = 0.001). aPLA were more frequently found in patients with cerebrovascular events vs other arterial thrombotic complications or no thrombosis, respectively (39.3, 6.1, and 12.9%; *p* < 0.001). MPN patients with thrombosis were more frequently positive with aPLA and had platelet receptor GP Ia/IIa c.807C>T CT/TT genotypes compared to MPN patients without thrombosis (18.6 and 2.0%; *p* = 0.006). aPLA alone or with coexistence with platelet receptor GP Ia/IIa c.807C>T CT/TT polymorphism could be associated with thrombotic complications in patients with MPN.

## 1 Introduction

*BCR-ABL*-negative myeloproliferative neoplasms (MPNs) – polycythemia vera (PV), essential thrombocythemia (ET), and primary myelofibrosis (PMF) – are grouped together within the WHO MPN category [1]. The most important *BCR-ABL*-negative MPN complications that interfere patient’s morbidity and mortality are thrombosis. They occur in all MPN entities range from 13 to 40% of patients [2,3,4].

The pathogenesis of thrombosis in MPN is multifactorial. Hematopoietic cell’s clonal proliferation, blood cells, and endothelial activation as well as close interactions of circulating plasma markers are known to take role in thrombosis pathogenesis in MPNs [5,6]. aPLA – anti-β2 glycoprotein 1 antibodies (anti-β2GP1) – bind to the β2GP1 receptors on the platelet surface, activating them that is important for thrombus formation [7]. In MPN patients, circulating platelets are already activated and they secret higher levels of surface P-selectin and tissue factor (TF) [8]. These processes allow thrombin formation on the platelet surface and initiation of thrombosis via thromboxane A2 synthesis [7]. Other cells are are activated as well: monocytes and endothelial cell. Recent data also suggest that neutrophils with TF expression and the release of neutrophil extracellular traps (NETosis) may contribute to aPLA-related vasculopathy in antiphospholipid syndrome (APS). Antiphospholipid syndrome is characterized by the presence of persistent aPL antibodies including anticardiolipin antibodies (aCL), lupus anticoagulant (LA) and anti-β2GP1 [8]. We included the analysis of anti-β2GP1 as in MPN patients, circulating platelets are already activated, and they secret higher levels of surface P-selectin and TF [9]. These processes allow thrombin formation on the platelet surface and initiation of thrombosis via thromboxane A2 synthesis [7]. Endothelial cells are additionally activated in MPN after aPLA interaction, and this process plays an important role in the pathogenesis of thrombosis [10].

There are limited data that analyzed aPLA in MPN patients. In 50 patients of Jensen et al.’s cohort, immunoglobulin M (IgM)–aCL were detected in 22% of MPN patients and only 3% of the control group [11]. Bidot et al. found at least one positive aPLA in 66% of tested subjects with ET. The incidence of aPLA in patients with thrombotic complications was 92.8% for those with thrombosis and 47.6% for those without thrombosis [12].

In the prospective study of 1,179 patients, conducted by Schwarz et al., aPLA were significantly associated with thrombotic complications [13].

In a study of 192 patients with MPNs, Tevet et al. identified aPLA (LA) in nine patients (14.51%), which were significantly related to thromboses [14].

The main purpose of our study was to evaluate the antiphospholipid antibodies (aPLA) and possibly their association with thrombosis as well as in their possible relationship with other risk factors in patients with MPNs. We also investigated combined aPLA effect on thrombotic complications together with the platelet receptor glycoprotein (GP) Ia/IIa c.807C>T CT/TT polymorphisms at the Department of Oncology and Hematology of the Institute of Oncology, the Lithuanian University of Health Sciences.

## 2 Methods

### 2.1 Samples and baseline

The study was conducted at the Department of Hematology of the Institute of Oncology, the Lithuanian University of Health Sciences. It included 108 patients with *BCR-ABL*-negative MPN. The diagnosis of ET, PV, and PMF was according to the WHO 2016 diagnostic criteria. Data of previous thrombosis and risk factors as well as clinical and laboratory findings were collected. Arterial or venous thrombosis, such as stroke, myocardial infarction, transient ischemic attack, unstable angina, legs’ deep vein thrombosis, legs’ superficial vein thrombosis, thrombosis of abdominal veins, and thrombosis of the pulmonary artery (PE) were defined as vascular events. Those complications were assessed with reference to medical documentation.

There were two different selective and one confirmatory tests for the aPL determination. Tests were performed at the Laboratory Clinic of Kaunas Clinics of the Lithuanian University of Health Sciences Hospital.

The genomic DNA was isolated from peripheral blood leukocytes using the DNA-purification kit according to the manufacturer’s standard protocol at the Oncology Research Laboratory, Institute of Oncology. For all patients included in the study, genotyping of platelet receptor GP Ia/IIa (c.807C>T) polymorphism was performed. Results of other platelet receptor GP polymorphism were published before [15].

All comparisons were performed between two groups of MPN patients – who experienced thrombosis and who did not experience thrombosis. Informed consent has been obtained from patients who participated in clinical investigations.


**Informed consent:** Informed consent has been obtained from all individuals included in this study.
**Ethical approval:** The research related to human use has been complied with all the relevant national regulations, institutional policies, and in accordance with the tenets of the Helsinki Declaration and has been approved by the authors’ institutional review board or equivalent committee.

### 2.2 Statistical analysis

Statistical analyses were performed with IBM SPSS Statistics 22 (SPSS Inc., Chicago, IL, USA). Categorical (qualitative) variables were compared using the Pearson chi-square feature independence (homogeneity) criterion. The results are described by the frequencies and relative frequencies (percent) of the significance values of the traits. Quantitative characteristics that satisfied the conditions of the normal partition were compared in independent groups using the Student’s *t* criterion and those that did not meet the Mann–Whitney *U* criterion. The data are described in the first case with mean (standard deviation [SD]) and in the second case with median (minimum - maximum values; means). All statistical tests were two-sided, and the level of statistical significance was established at *α* = 0.05 (*p*-value <0.05).

## 3 Results

In the study population of 108 patients, 60 (55.6%) patients were diagnosed with ET, 41 (38%) patients were diagnosed with PV, and 7 (6.5%) were PMF patients. Demographic data of the patients were published earlier [15]. The *JAK2* V617F mutant allele was present in 71.4% of PMF patients, 87.8% of PV and 61.7% of ET patients. Thrombotic complications were present in 59 (54.6%) subjects of those with MPN. The patients with thrombotic complications were older than those without thrombotic complications, 66.98 (SD = 13.42) years and 60.17 (SD = 15.58) years (*p* = 0.016). There were no gender differences between the groups. LA was present in nine (8.3%) MPN patients. Solid phase aPLA were detected in 18 (16.7%) MPN patients. Patients with positive aPLA were older than those without aPLA (72.5 and 63.0 years; *p* = 0.017). PV patients were more likely to have aPLA than ET or PMF patients (66.7, 27.8, and 5.6%; *p* = 0.6). Distribution of different aPLA classes in MPN patients with thrombosis and without thrombosis is shown in Table 1.

**Table 1:** Distribution of aPLA classes in MPN patients with thrombosis compared to patients without thrombosis

aPLA	No thrombosis, *n* (%)	With thrombosis, *n* (%)	*p* value
aCL IgM	1 (2.0)	3 (5.1)	0.41
aCL IgG	—	8 (13.6)	—
anti-β2 GPI IgM	1 (2.0)	2 (3.4)	0.67
anti-β2 GPI IgG	2 (4.1)	7 (11.9)	0.14

aPLA – antiphospholipid antibodies; aCL – cardiolipin antibodies, anti-β2 GP1 – anti-glycoprotein 1 antibodies.

**Figure 1:**
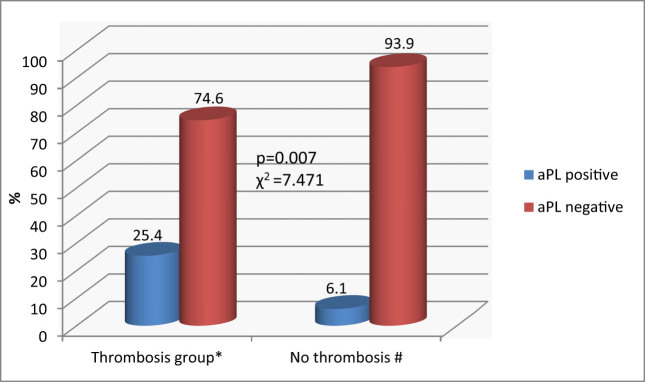
aPLA in MPN patients (*n* = 108). *χ* ^2^ – chi-square test. *Data are presented as % in the thrombosis group. ^#^Data are presented as % in the no thrombosis group.

aPLA were significantly more frequently found in MPN patients with thrombosis than in patients without thrombosis (25.4 and 6.1%; *p* = 0.007) (Figure 1).

**Figure 2:**
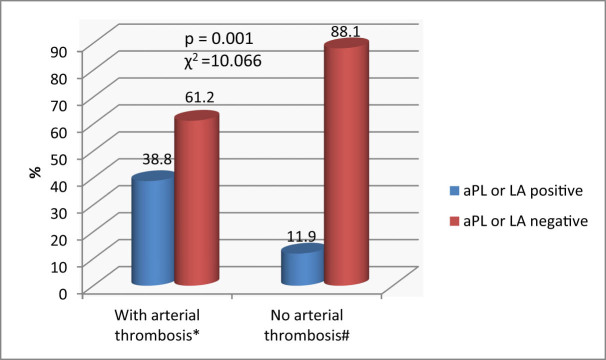
aPLA and LA in MPN patients (*n* = 108). *χ* ^2^ – chi-square test. *Data are presented as % within thrombosis group patients. ^#^Data are presented as % within group of patients without thrombosis.

MPN patients with arterial thrombotic complications were significantly more frequently positive for aPLA compared to those without arterial thrombosis (38.8 and 11.9%; *p* = 0.001) (Figure 2).

**Figure 3:**
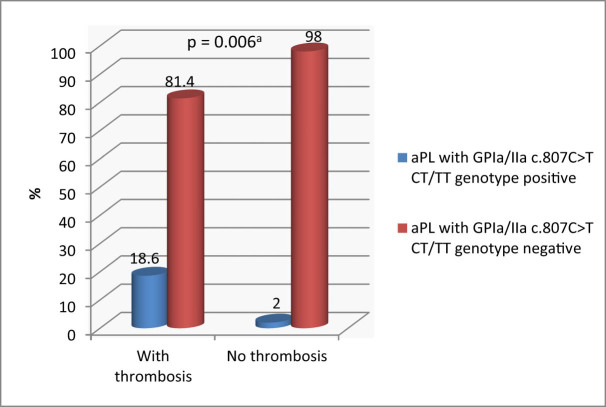
Distribution of the aPLA together with platelet receptor GP Ia/IIa c.807C>T CT/TT genotypes in MPN patients (*n* = 108). ^a^Fisher‘s exact test. *Data are presented as % in the thrombosis group. ^#^Data are presented as % in the no thrombosis group.

MPN patients with thrombotic complications were significantly more frequently positive with aPLA and had platelet receptor GP Ia/IIa c.807C>T CT/TT genotypes compared to MPN patients without thrombotic complications (18.6 and 2.0%; *p* = 0.006) (Figure 3).

aPLA were significantly more frequently found in patients with cerebrovascular events (ischaemic stroke and TIA) compared to MPN patients with other thrombotic complications or MPN patients with no thrombosis, respectively (39.3, 6.1 and 12.9%; *p* < 0.001).

## 4 Discussion

The main objective of this study was to evaluate whether aPLA could be associated with the risk of thrombosis alone or with the other risk factors in patients with MPNs.

Circulating aPLA and lupus anticoagulant are found in patients with malignancies, in APS and in patients with arterial and venous thrombosis [16]. It is known that the formation of aPLA on the cell membrane affects them not only directly but also via various cofactors, such as platelet receptor beta-2 (β2) glycoprotein-1 [10].

In our patient’s blood samples, we found a higher incidence of aPLA in older patients than in younger patients, and this was consistent with data reported in the literature [17].

Analyzing data from patients with different MPN, we found that aPLA is more commonly detected in PV than in patients with ET or PMF, although this difference was not statistically significant in our research. Bidot et al. also found a relationship between thrombotic complications and aPLA in ET patients [12]. In the study of the Schwarz et al., aPLA were significantly related to thrombosis [13]. Our study found that the aPLA increased the risk of thrombotic complications.

When we separated patients with arterial thrombosis according to different arterial circulation regions, we found that aPLA were statistically significantly more common in patients with ischemic stroke and TIA than in subjects who had not experienced thrombotic complications or experienced other thrombotic complications. Djokovic et al. found that APS subjects who experienced cerebrovascular thrombotic complications were more likely to be diagnosed with IgG β2GP1 antibodies than those who had not experienced these complications [18]. Carmel-Neiderman et al. who studied patients with the stroke and aPLA compared these data with the healthy control group. They also found a tendency for IgG-class aPLA to be more common in the ishaemic stroke group than in healthy people [19]. Meta-analysis that was done by Sciascia et al. included 5,215 patients from 43 clinical trials; it was found that the presence of aPLA increased the risk of ishaemic stroke and TIA in subjects under 50 years of age [20]. Our results support the data that aPLA could be associated with developing ischemic stroke and TIA in patients with MPN.

Analyzing the separate classes of aCL and anti-β2GPI, we found that IgG-class aCL and anti-β2 IgG aPLA were found more frequently than anti-β2 IgM aPL antibodies or aCL in patients with thrombotic complications.

In our research, we analyzed the synergism of aPLA antibodies and platelet receptor SNP effect on thrombosis. We described earlier platelet receptor and coagulation factor SNP impact on thrombosis [15]. Analyzing MPN subjects who had experienced thrombotic complications, we found that these subjects were more likely to be positive for aPLA antibodies in combination with GP Ia/IIa c.807 CT or TT genotype (T allele) than subjects without thrombosis. The c.807C>T T allele of the platelet receptor GP Ia/IIa (ITGA2 gene) has been shown to be associated with higher receptor density and an increased risk of thrombotic development [21]. On this basis, it can be assumed that platelets with this SNP are more active, while concomitant aPLA antibodies may activate them even more by binding to the platelet, creating favorable conditions for thrombosis. Therefore, we can hypothesize that these patients with MPN who are positive with aPLA and have platelet receptor GP Ia/IIa c.807 CT or TT genotype may be linked with more frequent thrombotic complications.

The results of our study show that aPLA alone or in combination with the platelet receptor GP Ia/IIa c.807 CT or TT genotype increased the risk of thrombotic complications in patients who are diagnosed with MPNs.
